# Cytotoxicity of Commercially Pure Titanium (cpTi), Silver-Palladium (Ag-Pd), and Nickel-Chromium (Ni-Cr) Alloys Commonly Used in the Fabrication of Dental Prosthetic Restorations

**DOI:** 10.7759/cureus.31679

**Published:** 2022-11-19

**Authors:** Passent Ellakany, Maram A AlGhamdi, Turki Alshehri, Zakia Abdelrahman

**Affiliations:** 1 Substitutive Dental Sciences, College of Dentistry, Imam Abdulrahman Bin Faisal University, Dammam, SAU; 2 Dentistry, College of Dentistry, Imam Abdulrahman Bin Faisal University, Dammam, SAU; 3 Immunology, Medical Research Institute, Alexandria University, Alexandria, EGY

**Keywords:** corrosion, dental implants, biocompatibility, human gingival fibroblast, nickel-chromium, silver-palladium, commercial pure titanium

## Abstract

Introduction: The longevity of dental implants is affected by the ability to avoid any hypersensitivity or corrosive reactions in the oral cavity. The aim of the current study was to evaluate the cytotoxic effect of commercially pure titanium (cpTi), silver-palladium (Ag-Pd), and nickel-chromium (Ni-Cr) on human gingival fibroblast (HGF).

Methods: The sample size used was 10 discs from each alloy used with dimensions of 4x3mm. The HGF was derived from healthy patients subjected to gingivectomy procedures. Of the specimens, 50% were incubated in artificial saliva and the other half in Dulbecco's Modified Eagle medium (DMEM). The extract of each alloy in both media was collected and applied on HGF. After 24 hours the morphology of the HGF cells was examined to detect any apoptosis or cell death. Also, cell viability was evaluated by the use of a 3-(4,5-dimethyl thiazol-2-yl)-2,5-diphenyltetrazolium bromide (MTT) assay. Statistical analysis was performed using students' t-test and two-way ANOVA with a significance level of p<0.05.

Results: In the case of morphological examination of HGF and MTT assessment, only cpTi alloy specimens didn’t display any cytotoxic effect. Ni-Cr was the most cytotoxic alloy of the three. Also, MTT activities of all three alloys were decreased when they were incubated in artificial saliva.

Conclusion: cpTi exhibited the highest corrosion resistance in comparison to Ag-Pd and Ni-Cr alloys. Ag-Pd alloys showed acceptable resistance to corrosion that is due to the passivity effect. Also, artificial saliva increased the cytotoxic effect of the tested alloys more than DMEM.

## Introduction

Dental implants are considered one of the most conservative prosthetic treatment choices for replacing missing teeth. They are widely used in stabilizing dentures; replacing missing teeth with implant-supported single crowns or fixed partial dentures, in addition to anchorage function in orthodontics procedures. To achieve optimum functions of dental implants, they need to have specific characteristics such as strength, durability, biocompatibility, wear resistance, and affordable cost. Several casting alloys are currently used in the fabrication of these different implant-supported dental restorations [1,2].

In previous years, gold-based alloys were used as the alloy of choice in the fabrication of dental prosthetic restorations as they had several advantages such as ductility, inertness, smooth surface, optimum corrosion resistance, and ease of margin adaptability and burnishing. However, the high expenses of gold lead to a dramatic shift in using noble, base-metal, and titanium alloys to overcome this drawback. Among these alloys, titanium became the material of choice in manufacturing dental implants [1]. The use of pure titanium and its alloys has increased because of possessing positive features such as resistance to fracture, strength, biocompatibility, optimum osseointegration capacity, longevity, and relative affordability, in comparison to gold alloys [2].

In the case of using porcelain fused to metal restorations, a metal substructure should be used to provide a strong framework to support porcelain in order to withstand occlusal masticatory forces. Application of dental porcelain to silver palladium alloys (Ag-Pd) enhances the mechanical and biological properties of the restoration by increasing the fracture toughness, strength, corrosion resistance, and matching coefficient of thermal expansion, besides enhanced wettability and adhesion of porcelain to Ag-Pd alloy. The presence of silver metal in Ag-Pd alloy also provides an anti-bacterial effect as it inhibits bacterial reproduction in addition to destroying the bacterial cell wall [3-5]. These features increased the chances of using Ag-Pd alloys as an efficient substitute for gold alloys in fabricating dental prosthetic restorations [6].

The longevity of dental restoration is a critical feature that needs to be considered where it can be achieved by avoiding any hypersensitivity or corrosive reactions in the oral cavity [1,7]. Corrosion is a mechanism that is characterized by the liberation of ions and is magnified in the presence of an acidic pH environment, food, and infection, which are frequently occurring in the patient's oral cavity [8,9].

The presence of a metallic restoration in the patient’s mouth causes a mutual interaction between the patient’s living tissues and the dental restoration. This reaction results in the release of elements from the alloys which interfere with the biochemical and enzymatic cellular reactions, leading to necrosis of living cells in the oral cavity [10]. These interactions are dependent on the type of the material of the dental restoration, host defense, and contact duration between the alloy and the surrounding tissues, added to the occlusal masticatory forces [9,11]. The elements released from the dental alloys in the patient’s mouth may penetrate the oral epithelium, gingival tissues, gastrointestinal system, or lungs causing systemic side effects such as allergic reactions, neurological response, and rheumatic complications [11,12]. Previous studies reported the occurrence of hypersensitivity reactions in patients having dental restorations manufactured of alloys including nickel, cobalt, chromium, iron, and mercury [11]. But still, further studies are needed to evaluate the cytotoxicity of these alloys and their side effects on patients.

Assessment of dental alloys' cytotoxicity can be performed by the use of cell lines as permanent mouse fibroblasts (L-929, 3T3) or human epithelial cells (HeLa) in order to simulate in vivo conditions inside the patient's oral cavity [7,13,14]. However, recent articles have recommended the use of cultures from biopsies of specified tissues such as human gingival fibroblasts (HGF) as an indicator of cytotoxicity because they exhibit high differentiation levels similar to the patients’ living tissues [7,13].

The aim of the current study was to assess the cytotoxic effect of elements released from commercially pure titanium (cpTi) in relation to other dental alloys (Ag-Pd and Ni-Cr) on HGF by morphological examination and 3-(4,5-dimethyl thiazol-2-yl)-2,5-diphenyltetrazolium bromide (MTT) assay (Roche Diagnostics, Dubai, United Arab Emirates). The null hypothesis states that cpTi is the most biocompatible among the three alloys.

## Materials and methods

Three different alloys (cpTi, Ag-Pd, and Ni-Cr) were used in the current study to assess their cytotoxic effect on HGF (Table 1). The sample size was calculated based on assuming 80% study power and 5% alpha error. Thus, the minimum sample size was calculated to be 9 per group, increased to 10 to make up for laboratory processing errors (G*Power v3.1.9.2; Heinrich Heine University Düsseldorf) [15]. The total sample size used was 30 specimens (n=10*3 discs) with 10 specimens for each alloy. cpTi alloy bar (American Society for Testing and Materials (ASTM) grade 2, Nippon Steel Corporation, Tokyo, 100-8071, Japan) was sectioned by Elite BB25-1 lathe milling machine (4 mm in diameter and 3 mm in thickness), polished, and finished according to the manufacturer instructions. The discs of Ag-Pd (IPS d.SIGN® 53, Ivoclar Vivadent, Liechtenstein) and Ni-Cr alloys (IPS d.SIGN®15, Ivoclar Vivadent, Liechtenstein) were manufactured by the conventional casting method by the use of specially designed split mold for construction of standardized wax patterns with dimensions of 4 mm in diameter and 3 mm in thickness.

**Table 1:** Composition of casting alloys used in this study. ASTM: American Society for Testing and Materials

Alloy	Manufacturer	Trade name	Description
Commercially pure titanium	Nippon Steel Corporation. (Tokyo 100-8071, Japan)	cpTi: ASTM grade 2	Bar
			10 cm length x 4 mm diameter
Silver-palladium	Ivoclar Vivadent (Liechtenstein FL-9494 Schaan)	IPS d.SIGN® 53	6mm x 3mm x 2mm thickness
			Pd 53.8%, Ag 34.9%, In 1.7%, Sn 7.7%, Zn 1.2%, Pt<1%, Re<1%, Ru<1%, Li<1%
Nickel-chromium	Ivoclar Vivadent (Liechtenstein FL-9494 Schaan)	IPS d.SIGN® 15	Cylinder 3cm length x 6mm diameter
			Ni 58.7%, Cr 25%, Mo 12.1%, Si 1.7%, Fe 1.9%, Co<1%, Ce<1%

Sterilization of alloys

Specimens were washed with Alconax soap (Alconox, Inc., New York City, New York, United States) and a soft brush then rinsed twice with distilled water. Each disc was treated ultrasonically with 1 mL of 70% isopropyl alcohol (Sigma, Aldrich, Germany) for five minutes. Then discs were washed again with sterile distilled water to eliminate traces of alcohol and left to dry for 48 hours at 60°C in aseptic culture plates before performing the test.

Alloys’ extracts

After the specimens were disinfected as mentioned above, half of the discs were incubated in 0.5 ml of DMEM media while the other half was incubated in 0.5 ml of artificial saliva (pH 6.5; Sigma, Aldrich, Germany) in a sterile Eppendorf tube for 72 hours with shaking. Then alloys’ extracts were isolated in aseptic tubes. Gibco Dulbecco's Modified Eagle Medium (DMEM) (Thermo Fisher Scientific Inc., Waltham, Massachusetts, United States) and artificial saliva were separately used as blank controls to evaluate the effect of the two solutions on the viability of HGF cells, while Teflon specimens were used as a negative control to eliminate the effect of Teflon used during sterilization of the specimens. Also, Formalin wells were used as a positive control to be a reference for the structure of dead cells in the wells of tested alloys.

HGF

The current study was approved by the Institutional Review Board in Medical Research Institute, Alexandria University, (approval number 2019052), and informed consent was signed by the patients. HGF was obtained from the gingival tissue of healthy patients who underwent surgical gingivectomy in the premolar region. Gingival specimens were placed in DMEM including 15% fetal bovine serum (Gibco FBS; Cat. no. 10099141, Thermo Fisher Scientific, Inc.) with 100 U/mL penicillin G, 50 ng/mL amphotericin B, and 100 μg/mL streptomycin (Gibco; Cat. no. 10378016, Thermo Fisher Scientific, Inc.). Then the gingival specimen was sectioned into small segments and placed in the tissue culture flasks filled with DMEM media.

HGF specimen was developed as a single layer in T-75 flasks incubated in 5% CO2 at 37ºC. Separation of adhered cells in a logarithmic growth phase of 75% was performed by adding 2-3 mL of DMEM containing 0.05% trypsin and 0.02% ethylenediamine tetraacetic acid (EDTA; Gibco, Thermo Fisher Scientific, Inc.). This technique was repeated every five days where the culture medium was changed twice per week and the cells were isolated four to seven times. The number of HGF cells was counted and the percentage of viable cells was determined by the trypan exclusion test, dye exclusion test (DET), using 0.2 % trypan blue. The viability of cells counted in the assays was over 90%.

Cells were plated in 96-well plates at a density of 4000 cells per well in a 100 μL culture medium and were maintained for 24 hours in an incubator to resume exponential growth. Then the medium was removed from each well and replaced by 100 μL of the alloy extracts that were previously filtered in a membrane filter (Puradisc, 0.2-µm pore size syringe filter; Whatman, Clifton, New Jersey, United States). Control wells were treated with 100 μL DMEM or artificial saliva. Triplicate wells for each alloy extract were prepared.

After 24 hours, the morphology of the HGF cells in the control group and those exposed to different alloys’ extracts were examined for apoptotic cells (loss of cellular symmetry, presence of blebbing, cell detachment, and cell death). The blinded examination was done under an inverted microscope and apoptotic cells in five high-power fields were counted where the mean was calculated. A scoring system was used as follows: presence of >1 apoptotic cell= non-cytotoxic; 2-3 apoptotic cells= mildly cytotoxic; 3-4 apoptotic cells= moderately cytotoxic; >4 apoptotic cells= highly cytotoxic [16].

MTT colorimetric assay

After incubation of HGF cells in the alloys’ extracts, viability was assessed by MTT colorimetric assay. This test is based on the ability of mitochondrial succinate dehydrogenase (SDH) to convert the yellow MTT dye into insoluble blue formazan. Viable cell count is directly proportional to the quantity of formed formazan [17].

Three control wells of DMEM were used to provide blanks of absorbance readings. A quantity of 10 μL of MTT reagent was added to each well and then placed in a cell culture incubator for two to four hours. The cells were periodically viewed under an inverted microscope till a clear visible intracellular punctate purple precipitate of formazan particle was formed. About 100 μL of detergent reagent was added to all wells, including the controls. The plate was swirled gently and then left covered in the dark for two to four hours at room temperature.

The optical densities (OD) of the produced solutions were detected with a microplate reader at 570 nm. Average values were estimated from triplicate readings and a calculation of the relative MTT activity (% control) was done.

Statistical analysis

IBM SPSS Statistics for Windows, Version 28.0 (Released 2021; IBM Corp., Armonk, New York, United States) was used in statistical analyses where the means and standard deviations were calculated. Student t-test and two-way ANOVA were performed with a significance level set at p<0.05.

## Results

Morphological examination

**Figure 1:**
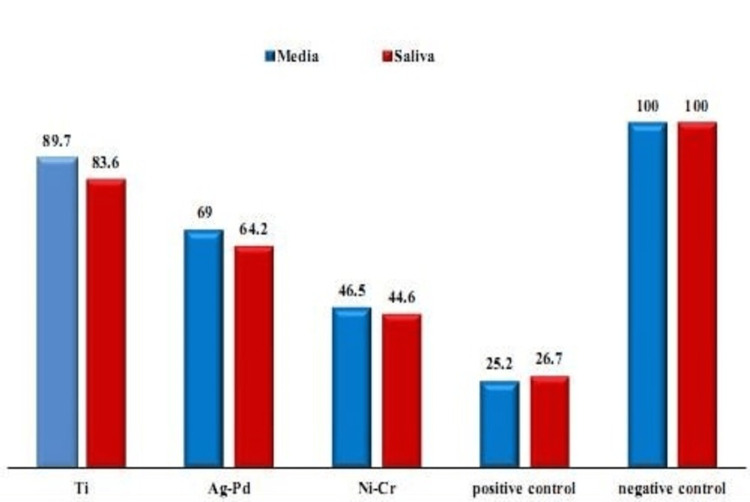
Effect of elements released from different alloys on HGF cell line. HGF: human gingival fibroblasts

Metal ions released from cpTi, Ag-Pd, and Ni-Cr alloys on both DMEM and artificial saliva caused detachment of HGF cells and loss of cellular symmetry. cpTi discs didn’t display any cytotoxic effect on HGF while Ag-Pd and Ni-Cr discs exhibited mild and moderate cytotoxic effects respectively according to the scoring system mentioned in the methodology (Figure 1).

MTT assay

**Figure 2:**
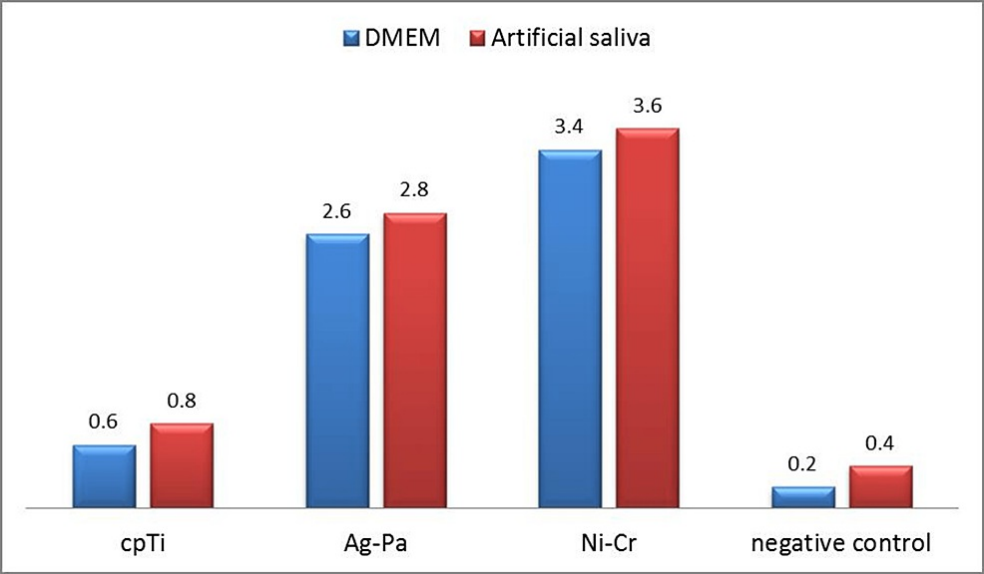
Percentage of mean metabolic activity (MTT) of HGF cell line with different alloys (relative to control: 100% metabolic activity). MTT: 3-(4,5-dimethyl thiazol-2-yl)-2,5-diphenyltetrazolium bromide; HGF: human gingival fibroblast; DMEM: Dulbecco's Modified Eagle Medium; cpTi: commercially pure titanium; Ag-Pa: silver-palladium; Ni-Cr: nickel-chromium

After incubating the alloys in DMEM, cpTi discs didn’t show significant reduction in MTT activity (mean (SD)=0.411(0.005), p=0.42) but in the case of Ag-Pd and Ni-Cr discs, the MTT activities were significantly reduced in comparison to control groups (mean (SD)=0.316 (0.006); p=0.01, mean (SD)= 0.213 (0.003); p=0.02 respectively). Ni-Cr specimens exhibited the highest reduction in MTT activity on HGF (Figure 2). The same effect was observed when incubating the alloys in artificial saliva, cpTi specimens exhibited the lowest MTT activity values but were not statistically significant (mean=0.38 (0.003); p=0.2), while Ag-Pd and Ni-Cr specimens exhibited statistically significant reduction in MTT activities (mean (SD)=0.29 (0.002); p=0.01, mean (SD)= 0.2 (0.003); p=0.02) respectively (Figure 2).

In comparing the cytotoxic effect of both DMEM and artificial saliva on the alloys used, the three alloys exhibited a statistically significant reduction in MTT activity of HGF cells in the case of artificial saliva. However, the alloys didn’t exhibit any significant cytotoxic effect on HGF cells when they were incubated in DMEM (Table 2).

**Table 2:** Comparison between elements released from different alloys in both artificial saliva and DMEM media regarding cytotoxicity (MTT) of HGF cells. P is statistically significant at < 0.05 MTT: 3-(4,5-dimethyl thiazol-2-yl)-2,5-diphenyltetrazolium bromide; HGF: human gingival fibroblast; DMEM: Dulbecco's Modified Eagle Medium; cpTi: commercially pure titanium; Ag-Pa: silver-palladium; Ni-Cr: nickel-chromium

MTT Absorbance at 492nm	Artificial Saliva	DMEM media	P-value
	Mean (SD)	Mean (SD)	
cpTi	0.383 (0.003)	0.411 (0.005)	0.013*
Ag-Pd	0.294 (0.002)	0.316 (0.006)	0.015*
Ni-Cr	0.204 (0.003)	0.213 (0.003)	0.05*

## Discussion

The release of metal ions from any metallic restoration in the oral cavity is usually provoked by several factors such as differences in temperature and pH values, the presence of bacteria, and the type of ingested food [18]. The current study evaluated the cytotoxic effect of metal ions released from three different dental alloys (cpTi, Ag-Pd, and Ni-Cr) commonly used in the fabrication of prosthetic restoration. The findings of the current study showed that cpTi didn’t exhibit a cytotoxic effect in morphological examination in addition to the lowest MTT activity among both DMEM and artificial saliva incubation when compared to Ag-Pd and Ni-Cr alloys. Therefore, the null hypothesis was accepted as cpTi discs showed the least cytotoxic effect in comparison to Ag-Pd and Ni-Cr discs respectively.

Our results showed that metal ions released from cpTi didn’t show any significant reduction in the MTT activity of HGF cells. However, metal ions released from Ni-Cr showed more reduction of MTT activity in comparison to those of Ag-Pd discs where both were statistically significant. Metal ions released in DMEM didn’t exhibit a significant reduction in MTT activity unlike those liberated in artificial saliva. DMEM was used to evaluate the corrosive effect of proteins and other molecules on the cytotoxic effect of the casting alloys to simulate dental restorations placed subgingivally away from exposure to natural saliva. Also, artificial saliva was used to evaluate the corrosive effect of minerals naturally available in the oral saliva simulating dental restorations in direct contact with the patient’s saliva.

The HGF cell line was used in the current study as the HFG cell line has the ability to maintain several differentiated features similar to the patient’s oral conditions [7]. The precise sensitivity of the MTT assay helped in detecting the occurrence of cytotoxic reactions by depending on the cell mitochondrial activity in comparison to lactate dehydrogenase (LDH) leakage and protein assays [17].

The current results were in agreement with Barrak et al., who found that pure titanium alloys (cpTi) exhibited better cell viability after 10 days of incubation in DMEM and no cytotoxic effect was noticed in the direct contact of alloy with cells unlike the results of Ti-6Al-4 V alloys [19]. This reduced cytotoxic effect of cpTi might be related to the low amount of vanadium ions released that leads to cell necrosis [20]. Additionally, another article showed better cell viability than that of Ti-6Al-4V alloys on HGF after both 48 and 72 hours, which referred to the liberation of aluminum and vanadium ions from the latter alloy causing cell death. But the release of ions was stabilized and reduced over time for both alloys. This was explained by the formation of the TiO passive layer limiting the release of ions from titanium alloys [21]. Cardoso et al. also found that pure titanium alloys used in combination with molybdenum (Mo) and niobium (Nb) elements showed no cytotoxicity effect as the result of cpTi alone [22]. 

Sun et al.'s findings were similar to the current findings as they found that both Ag-Pd and Au-Pd alloys had similar corrosion resistance [6]. This is referred to as the high amount of Pd in Ag-Pd alloy exceeding 50% and less than 90% of the alloy composition. Moreover, the presence of silver reduces the corrosion tendency of Ag-Pd alloy. Similarly, another research noticed higher cell viability with Ag-Pd alloys under static and dynamic loading tests in comparison to cobalt-chromium (Co-Cr) alloy, which was attributed to the high content of Pd that had corrosion resistance function [23].

In the case of the Ni-Cr cytotoxicity, other studies found that Ni ions released from Ni-Cr alloy exhibited the highest cytotoxic effect and this was referred to the high percentage of nickel in Ni-Cr alloy, unlike the Co-Cr alloys that exhibit passivity feature by forming oxide layer of chromium reducing the corrosion behavior. This toxicity level was enhanced by repeated times of alloy recasting [24]. In addition, the formation of the NiO layer on the alloy enhances Ni ions dissolution, especially at an acidic pH, causing it to be more corrosive [10,14]. These findings were similar to the current results. Recently, different digital methods of manufacturing Ni-Cr alloys were introduced in the dental market to improve cell viability in relation to the conventional method of fabrication [25].

Therefore, the high biocompatibility of cpTi in this study supported its use in the fabrication of dental implants, metallic denture frameworks, and metallic coping for porcelain fused to metal restorations than Ni-Cr alloys as Ti forms an oxide layer that prevents the dissolution of ions unlike Ni in Ni-Cr alloy [26,27].

The effect of saliva on metallic ion release agreed with the findings of several articles that found a decrease in the elemental release of the casting alloys in the cell culture medium than in saline-bovine serum albumin (BSA) while the least release was noticed in pure saline [28-30]. This might be due to the difference in composition of artificial saliva from the culture medium that had a great impact on the viability of the cells in the cell culture. Also, saliva enhances the dissolution of metal ions from dental alloys, especially in the presence of acidic pH or environment leading to more corrosive reactions [18].

The current in vitro study was limited to the use of three different dental alloys studied in a neutral pH environment. Thus, further studies need to be done to assess the cytotoxicity of other types of alloys used in dentistry. Also, both acidic and alkaline conditions should be tested to evaluate the effect of pH value on the cytotoxicity of alloys. Moreover, in vivo experiments should be performed to simulate the conditions of the oral environment such as variation in temperature, masticatory forces, and interaction between different metallic restorations in the oral cavity.

## Conclusions

Based on the study limitations, it was found that cpTi alloy exhibited the highest resistance to corrosion and passivity in comparison to Ag-Pd and Ni-Cr alloys in both artificial saliva and DMEM solutions. In the case of Ag-Pd alloys, they showed acceptable corrosion resistance values due to the presence of high palladium content and silver ions that enhance biocompatibility and the antibacterial effect. In viewing the direct contact of alloys with the living cells, titanium showed the least cytotoxicity unlike Ag-Pd and Ni-Cr alloys. The toxic effect of dental alloys was increased in the presence of artificial saliva than that in DMEM culture media due to the presence of minerals in saliva that increases the corrosive effect. Thus, the optimum dental alloy for use would be titanium alloys to fabricate dental implants, fixed partial dentures, and removable partial dentures and provide biocompatible dental appliances with proper longevity in the patient's oral cavity.
